# Relationship between the Gene Expression of Adenosine Kinase Isoforms and the Expression of CD39 and CD73 Ectonucleotidases in Colorectal Cancer

**DOI:** 10.32607/actanaturae.11871

**Published:** 2023

**Authors:** G. A. Zhulai, M. I. Shibaev

**Affiliations:** Institute of Biology, Karelian Research Centre, Russian Academy of Sciences, Petrozavodsk, 185910 Russian Federation; Endoscopic Department, Baranov Republican Hospital, Petrozavodsk, 185910 Russian Federation

**Keywords:** adenosine kinase, ADK-S, ADK-L, CD39, CD73, CD8+ T cells, CD4+ T cells, Treg cells, colorectal cancer

## Abstract

Tumor cells have the capacity to create an adenosine-rich immunosuppressive environment, which can interfere with antitumor immunotherapy. Approaches are currently being developed with a view to suppressing the production of adenosine or its signals. Such approaches include the use of antibodies to inhibit CD39, CD73, and adenosine-receptor antagonists. However, the abundance of enzymatic pathways that control the ATP-adenosine balance, as well as the still poorly understood intracellular adenosine regulation, makes the hoped-for success unlikely. In the present study, the enzyme adenosine kinase (ADK) needed to convert adenosine to adenosine monophosphate, thereby regulating its levels, was investigated. To do so, peripheral blood samples from patients with colorectal cancer (CRC) (*n *= 31) were collected with blood samples from healthy donors (*n *= 17) used as controls. ADK gene expression levels and those of its long (*ADK-L*) and short (*ADK-S*) isoforms were measured. The relationship between the levels of *ADK *gene expression and that of *CD39*, *CD73*, and *A2aR *genes was analyzed. It turned out that in the group of CRC patients (stages III-IV), the level of *ADK-L *mRNA was lower (*p* < 0.0011) when compared to that of the control. For the first time, an average correlation was found between the level of expression of *CD39 *and *ADK-S *(*r *= -0.468 at *p *= 0.043) and between *CD73 *and *ADK-L *(*r *= 0.518 at *p *= 0.0232) in CRC patients. Flow cytometry was used to assess the content of CD39/CD73-expressing CD8^+^, CD4^+^ and Treg lymphocytes, as well as their relationship with the level of ADK gene expression in CRC patients. But no significant correlations were found.

## INTRODUCTION

The role of extracellular adenosine in the tumor microenvironment has been sufficiently researched [1]. Adenosine can regulate the innate and adaptive immune responses [2] by inhibiting the activity of the effector component and stimulating the immunosuppressive component. Thus, extracellular adenosine acts as a barrier for antitumor immunotherapy. The therapeutic potential of enzyme blockade, specifically that of the ectonucleotidases CD39 (ectonucleoside triphosphate diphosphohydrolase, ENTPD1) and CD73 (ecto-5’-nucleotidase, 5’NT) involved in ATP breakdown to adenosine and inhibition of adenosine receptors (primarily A2a) was demonstrated in preclinical trials and is now being tested in oncological patients in clinical trials I/II [3]. However, the hoped-for efficacy, based on preclinical trials, is yet to be achieved [4].

Numerous pathways controlling the ATP-adenosine balance still remain understudied. Approaches to the blockade of the adenosine signaling pathway are usually developed with little attention paid to the intracellular adenosine regulation. Aside from the “classical” extracellular adenosine synthesis pathway from ATP by ectonucleotidases CD39-CD73, recent discussions have tended to focus on the role of the alternative pathway involving extracellular nicotinamide adenine dinucleotide (NAD^+^) in cancer progression [5]; so, a study into other adenosine metabolism components in developing tumors seems relevant.

The adenosine content is regulated by adenosineconverting enzymes; i.e., adenosine kinase (ADK) and adenosine deaminase [6, 7]. ADK adds a phosphoric acid residue to adenosine and converts it into AMP. Adenosine deaminase removes amino groups from adenosine molecules, with inosine as a by-product. On top of that, the adenosine level may be regulated by the way it is delivered to the extracellular space by bidirectional nucleotide transporters.

Of special interest here is adenosine kinase regulating the availability of adenosine while also being involved in complex homeostatic and metabolic networks [8]. The balance between adenosine and ADK is strictly maintained in healthy cells, while changes in enzyme expression lead to various degrees of activation of adenosine receptors, which often determines the role of ADK in the development of the pathology [9]. Apart from purine metabolism, ADK is also involved in the regulation of transmethylation. A relationship between ADK expression and DNA methylation has also been demonstrated. The use of specific ADK inhibitors may reduce the global DNA methylation level in HeLa cells in a dose-dependent fashion [10]. Human ADK is represented by two isoforms with different molecular masses and, presumably, functions. The short isoform ADK-S localized in the cytoplasm ensures routine metabolic removal of adenosine under normal conditions by means of its phosphorylation into AMP. The key function of ADK-S is to regulate the level of extracellular tissue adenosine. The long isoform ADK-L localized in nuclei has a direct biochemical link to S-adenosylmethioninedependent transmethylation pathway-controlling DNA and histone methylation. High levels and degree of activity of ADK-L are associated with increased global DNA methylation [1].

The role of ADK in carcinogenesis is poorly studied. The available data [10, 11, 12, 13, 14, 15] suggests that there is a potential role for ADK in the development of colorectal cancer (CRC) [14], as well as breast [15] and liver cancer [12]. Other evidence of possible ADK involvement in tumor development includes the relationship between ADK and angiogenic activity and cellular proliferation during ontogenesis, as well as the changes in ADK expression in tumor tissue and its association with epigenetic regulation [8].

CRC is a common malignant disease and a major cause of cancer-related deaths. The adenosinergic pathway closely related to adaptive immunity suppression plays a significant part in CRC pathogenesis [16]. However, the relationship between ADK and immune mechanisms in CRC has not been properly studied. Given that, the goal of the present paper was to study the mRNA levels in *ADK, ADK-L*, and *ADK-S *and their relationship to the contents of CD39/CD73-expressing T cells in the peripheral blood of CRC patients.

## EXPERIMENT

The test material included vein blood samples collected into tubes with K3EDTA. In the present study, 31 blood samples from patients with colon adenocarcinoma 65 ± 12.4 years were analyzed. All patients were diagnosed through clinical investigations with histological confirmation. Their clinical characteristics are presented in *Table 1*. The inclusion criteria were age of over 18 and large colon cancer as a confirmed diagnosis. The exclusion criteria were neoadjuvant therapy administration and reports of autoimmune and inflammatory diseases in the recent three months. We also analyzed 17 blood samples from healthy donors of comparable age (56.10 ± 17.70) as the controls. The study was carried out in compliance with the requirements of the Declaration of Helsinki and approved by the Medical Ethics Committee of the Ministry of Healthcare and Social Development of the Republic of Karelia and Petrozavodsk State University (protocol No. 25 dated February 12, 2013). All participants gave their informed consent in writing prior to inclusion in the study.

**Table 1:** Patient characteristics

Parameter		CRC patients	Healthy donors
Sample size		31	17
Sex	M	11 (35.5%)	6 (35.3%)
	F	20 (64.5%)	11 (64.7%)
Median age (min–max)		65.0 (45–78)	55.0 (28–79)
CRC stage	1–2	16 (51.6%)	–
	3–4	15 (48.3%)	
Tumor grade	G1	3 (9.7%)	–
	G2	23 (74.2%)	
	G3	5 (16.1%)	

**Gene expression analysis**

The total RNA was isolated from the blood using TRIzol LS reagent (ThermoFisher Scientific, the United States), DNA contamination was removed, and the samples were treated with DNase I (Lucigen, the United States). The quantity and quality of the obtained RNA was assessed using SmartSpec Plus spectrophotometer (Bio-Rad, the United States). Synthesis of cDNA was performed using random hexaprimers and reverse transcriptase MMLV (Evrogen, Russia). Amplification of the cDNA and analysis of the amplification products with real-time PCR was run using the master mix with a SYBR Green I intercalating dye (Evrogen, Russia), in accordance with the manufacturer’s manual on the iCycler amplifier with an iQ5 optical system (Bio-Rad, the United States), in duplicates with no template control. Expression of the genes of interest was normalized to the expression of the reference gene *GAPDH*. The primers used for the expression assessment of the genes *ADK, ADK-L, ADK-S, A2AR, CD39*, and *CD73 *(Syntol, Russia) are presented in *Table 2*. The optimal annealing temperature was determined by temperature gradient setup. The protocol for *ADK, ADK-L, *and *ADK-S *was as follows: cDNA denaturation for 5 min, at 95°C; 40 cycles: denaturation at 95°C, 30 s; annealing at 61°C, 30 s; elongation at 72°C, 30 s. The protocol for *A2AR, CD39*, and *CD73* was as follows: cDNA denaturation for 5 min, at 95°C; 40 cycles: denaturation at 95°C, 30 s; annealing at 64°C, 30 s; elongation at 72°C, 30 s. PCR specificity was controlled by analyzing melting curves. Relative gene expression levels were calculated using the 2–∆∆ Ct method, where Ct is the threshold cycle and ΔCt is the difference between the threshold cycle values for the reference and target genes. The total gene expression level was calculated with respect to the control (healthy donors), with the expression level of each gene of interest in the control taken as 1. The data are presented in per-unit notation and calculated as the mean value ± standard error (M ± SE).

**Table 2:** The nucleotide sequences of the primers used in this study

Gene	Primer ’ → 3’	
	Forward	Reverse
ADK	TTACTACGAGCAGAATGAGCAG	TGGCAGCAGCAAGATTAGC
ADK-L	TGTAGAGCCAAAGTGGGGTG	GCCTCCACCTTCAGCTTTTTG
ADK-S	AAGCAGTTGCTGTGGTACCTG	AGCAGAGGATTTCCCATTCCA
A2AR	CTTGGGTTCTGAGGAAGCAG	CAGCAGCTCCTGAACCCTAG
CD39	AGCAGCTGAAATATGCTGGC	GAGACAGTATCTGCCGAAGTCC
CD73	ATTGCAAAGTGGTTCAAAGTCA	ACACTTGGCCAGTAAAATAGGG
GAPDH	GGTGGTCTCCTCTGACTTCAACAG	GTTGCTGTAGCCAAATTCGTTGT

**Flow cytometry**

The whole blood samples were stained with antibodies and incubated for 20 min at room temperature in the dark in accordance with the manufacturer’s protocol. RBCs were lysed by BD FACS Lysing Solution (BD Biosciences, the United States). In the present study, the following monoclonal antibodies were used: CD3-PC5 (UCHT1 clone), CD4-FITC (OKT4 clone), CD4-PC7 (OKT4 clone), CD8-PC7 (RPA-T8 clone), CD25-PC5 (BC96 clone), CD127-PC7 (EBIORDR5 clone), CD73-PE (AD2 clone), CD39-PE (EBIOA1 clone), CD39-FITC (EBIOA1 clone) (eBioscience, the United States), as well as the respective isotype controls. All events were acquired using a Cytomics FC500 cytometer (Beckman Coulter, the United States). At least 30,000 events per sample were analyzed in the lymphocyte gate based on forward and side scatter. The data were presented as M ± SD.

**Statistical analysis**

The statistical processing and parameter calculation were performed using the GraphPad Prism v.7 software. The significance of the differences between the quantitative parameters was calculated using the nonparametric Mann–Whitney test. The differences were considered significant at *p * < 0.05. The correlation between parameters was estimated using Spearman’s test.

## RESULTS

**Expression level of adenosine kinase mRNA in the peripheral blood in CRC patients**

**Fig. 1:**
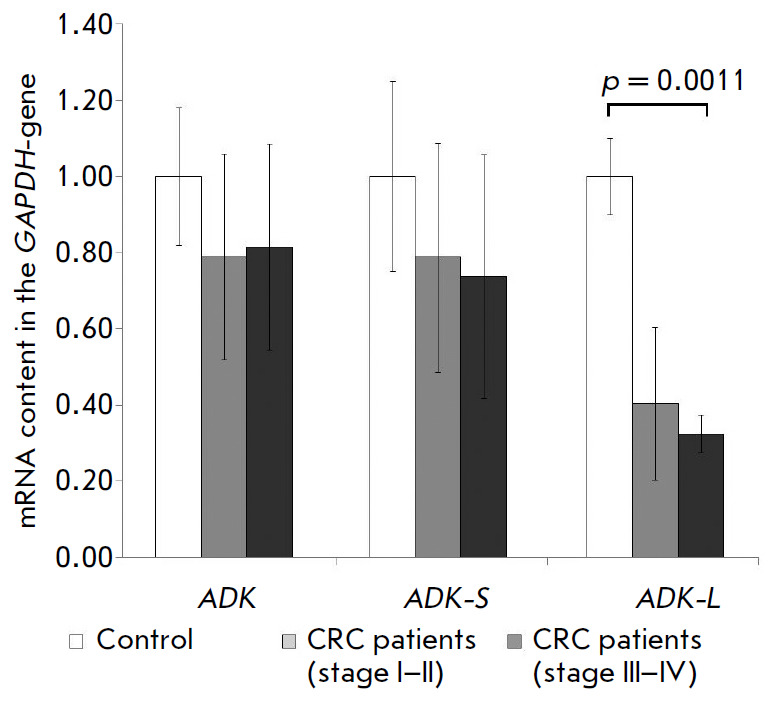
Changes in the relative level of mRNA of the *ADK*,* ADK-S*, *ADK-L *genes in the peripheral blood leukocytes of CRC patients if compared to healthy donors. The relative level of control mRNA was taken as 1. The normalization was performed according to *GAPDH*-gene mRNA

The data on ADK gene expression in CRC tissue are available in the literature [14], but it is yet to be studied how the ADK gene and its isoforms are expressed in the peripheral blood of CRC patients and how it is related to the clinical signs of the disease. We have estimated the relative content of mRNA of the* ADK *gene and its isoforms in the peripheral blood of CRC patients. The comparison of CRC patients with healthy donors showed a reduced *ADK-L *mRNA level (*p *= 0.002) in CRC. No differences from the control group were observed in the mRNA contents for the *ADK *gene and *ADK-S *isoform. Blood samples from patients with CRC stages III–IV showed reduced* ADK-L *mRNA levels (*p * < 0.001) as compared to healthy donors (*Fig. 1*). Meanwhile, no significant differences were observed in *ADK-L *mRNA levels between patients at early stages (I–II) and the control group. Finally, mRNA contents for the *ADK *gene and *ADK-S *isoform in the blood samples of CRC patients at both early and late stages were close to those in healthy donors.

In this study, the relationship between the mRNA levels of the gene of interest and the clinical signs of the disease was analyzed. A moderate negative correlation was established between the *ADK-L *mRNA content and tumor size (T2–T4), with a value of 0.508 at *p *= 0.038. However, no significant correlation was found between the *ADK-L *mRNA level and the disease stage. The differences in the *ADK *mRNA levels in CRC patients with and without distant metastases (M0–M1) or metastases to regional lymph nodes (N0–2) were not statistically significant.

**Table 3:** Correlation coefficient values between mRNA levels for the ADK gene and its isoforms ADK-S and ADK-L and mRNA levels for the CD39, CD73, and A2AR genes in CRC patients

mRNA level	ADK		ADK-S		ADK-L	
	r_S_	p	r_S_	p	r_S_	p
A2AR	-0.284	0.21	0.02	0.9346	0.406	0.0843
CD39	-0.038	0.097	-0.468	0.043	-0.329	0.168
CD73	-0.033	0.889	-0.16	0.511	0.518	0.0232

Note. Statistically significant parameters are highlighted in bold.

The extracellular adenosine level is regulated by the enzyme network, with the CD39 and CD73 ectonucleotidases playing a major part in carcinogenesis [17]. It has been demonstrated that the peripheral blood of CRC patients shows an increased *CD39* mRNA level, whereas the *CD73 *mRNA level remains the same as that in healthy donors [18]. We have analyzed the relationship between the relative expression of the genes *CD39, CD73*, and *A2AR *and the expression of the *ADK *gene and its isoforms in the peripheral blood of CRC patients. This has yielded new data on a correlation between gene expression levels: a negative correlation appears to exist between the *ADK-S *and *CD39 *mRNA levels. A positive correlation was identified between the *ADK-L *and *CD73* mRNA levels (*Table 3*).

**Relationship between the *ADK *gene expression level and CD39+/CD73+ T cell content**

**Fig. 2:**
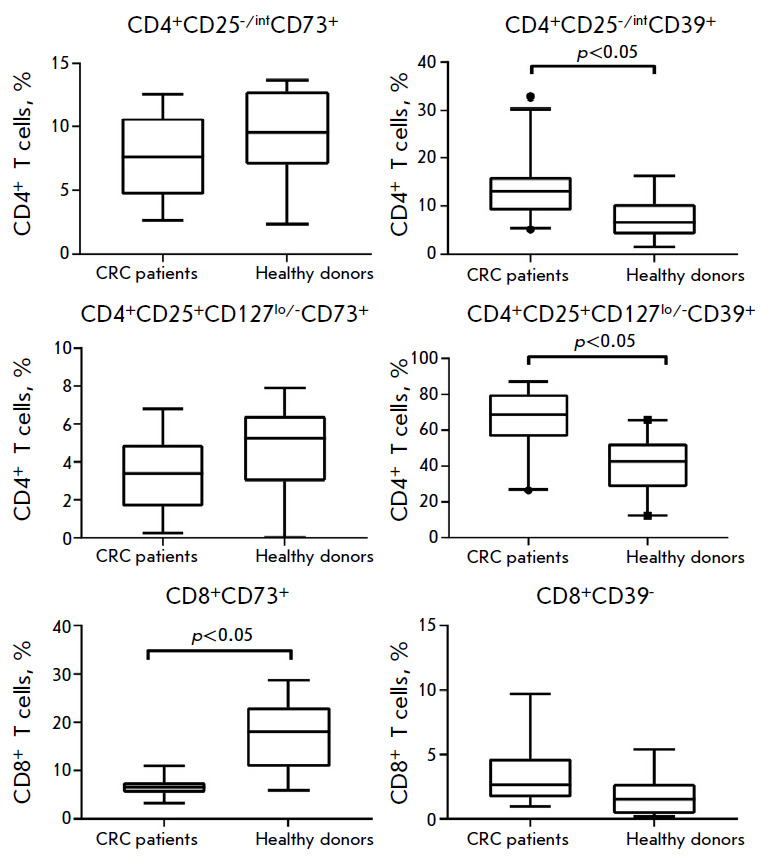
CD39^+^ and CD73^+^ T cells frequency in the peripheral blood samples of CRC patients and healthy donors

The established relationship between the mRNA levels for the ADK and CD39/CD73 ectonucleotidases in the peripheral blood implies that there is a relationship between ADK and CD39/CD73 expressing immune cells. The balance between CD8^+^ and CD4^+^ effector T cells and immunosuppressive regulatory T cells (Treg) is the key parameter of the antitumor immune response. Similarly to many other cells, these lymphocytes are sensitive to the adenosine effect primarily mediated by the A2aR adenosine receptor and may be involved in adenosine production through the expression of the CD39 and/or CD73 on their surfaces [3]. To probe for a relationship between the *ADK *expression level and the number of T cells involved in adenosine generation, the relative contents of CD39+/CD73+ effector T cells (CD4^+^ T helpers and CD8^+^ cytotoxic cells) and suppressive Treg cells were analyzed in CRC patients (*n *= 20) and healthy donors (*n *= 17) (*Fig. 2*).

**Fig. 3:**
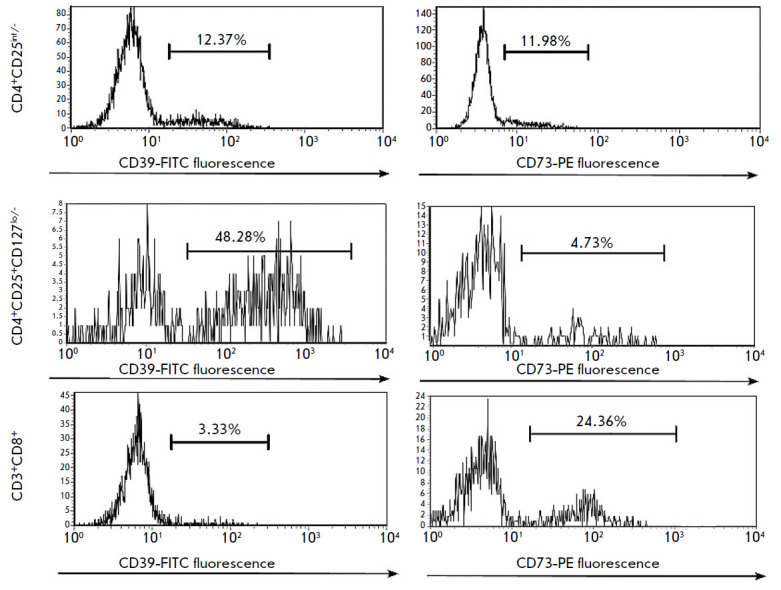
An example of CD39 and CD73 expression distribution histograms on the surface of CD8^+^ and CD4^+^ T cells in a healthy donor. The X-axis shows the fluorescence intensity of FITC and PE fluorochromes conjugated with antibodies against CD39 and CD73, respectively. The Y-axis shows the number of events in the lymphocyte gates. On the right, under the horizontal line, the cells expressing CD39/CD73 are marked; on the left are cells that are negative for CD39/CD73 expression

CD39-positive cells prevailed in Treg cells in both healthy donors and CRC patients, whereas CD73 expression was more characteristic of CD8^+^ T cells (*Fig. 3*). The same observations have been made by other authors [19]. Since the population of CD4^+^ effector T cells includes 3–% of Treg cells characterized by increased CD25 expression, the CD4^+^CD25-/int phenotype was analyzed to exclude the contribution of Treg cells to CD39/CD73 expression by T helpers.

It was discovered that about 64% of all Treg cells in the blood of CRC patients were CD39+, which was at significant variance with the Treg cell frequency in the blood of healthy donors (*p *= 0.0008), where CD39+ cells only accounted for 42% of all Treg cells. Significant differences were also observed for CD4^+^CD39+ T helpers (*p *= 0.037). The population of CD8^+^ T cells in CRC patients showed a reduced frequency of CD73-positive cells (*p *= 0.024). The frequency of CD73+ Treg cells, CD73+CD4^+^ T cells, and CD39+CD8^+^ T cells in CRC patients was no different from the control.

To estimate the correlation between ADK and the CD39+/CD73+ T cell frequency, we analyzed the possible relationships between the mRNA levels for *ADK* the gene and its isoforms *ADK-L *and *ADK-S *and the frequency of CD39/CD73-expressing T cells in the peripheral blood of CRC patients: no statistically significant correlations were found (*Table 4*).

**Table 4:** Correlation coefficient values between mRNA levels for the ADK gene and its isoforms ADK-S and ADK-L and relative contents of CD39^+^ and CD73^+^ T cells in the blood of CRC patients

T cells	ADK		ADK-S		ADK-L	
	r_S_	p	r_S_	p	r_S_	p
CD8^+^CD73^+^	0.107	0.840	0.178	0.713	-0.036	0.951
CD8^+^ CD39^+^	0.033	0.948	-0.217	0.581	0.126	0.295
CD4^+^CD25^-/int^CD73^+^	-0.217	0.581	-0.300	0.437	0.393	0.295
CD4^+^CD25^-/int^CD39^+^	-0.021	0.929	0.255	0.278	0.002	0.995
CD4^+^CD25^+^CD127^lo/-^CD73^+^	-0.381	0.359	-0.381	0.360	0.256	0.549
CD4^+^CD25^+^CD127^lo/-^CD39^+^	0.051	0.827	0.278	0.235	-0.151	0.522

## DISCUSSION

The adenosinergic pathway has gained in interest as a promising target for antitumor therapy. The key actors in this pathway – CD39/CD73/A2aR – show increased expression levels and activity in tumor tissue and are often associated with clinical signs of the disease and unfavorable prognosis in some cancer types [17]. Clinical trials produced a preliminary optimal safety profile for the A2aR and CD73 blockers and showed an increased overall response rate to them [4, 20]. Nevertheless, the positive results achieved through both monotherapy and combination therapy fell mostly below the expectations engendered by the pre-clinical trials. This is an indication that we need a more refined patient selection process or need to use biomarkers to better predict and optimize therapy results [4, 20].

The ADK enzyme regulates the adenosine level by converting it into AMP. We currently lack a clear understanding of the role of ADK in tumor development. Earlier papers show increased *ADK *gene expression levels [14] and enzymatic activity [21] in tumor tissue of CRC patients compared to healthy tissue. On the other hand, liver cancer patients show lower ADK protein levels compared to healthy tissue. In addition, a decrease in the ADK level in the liver resulted in higher sensitivity to the acute toxic effects of the carcinogen (diethylnitrosamine) in the experimental model [12]. Inhibition of tumor cell proliferation and induction of apoptosis after ADK inhibitor treatment, particularly in the colorectal cancer cell line HT-29, have been described in a series of experimental papers [22]. Information on the role of ADK isoforms in carcinogenesis is rather scarce. For instance, Shamloo et al. [15] have pointed to a more significant role for the long ADK isoform in breast cancer. The events caused by the respective gene knockdown point toward an involvement of this isoform in mitogenesis, carcinogenesis, and tumor cell invasion. ADK expression in peripheral blood and the relationship between ADK and the activation of the key lymphocyte populations associated with the antitumor immune response (CD8^+^/CD4^+^ T cells and Treg cells) in CRC patients remains poorly studied.

The results obtained in the present study confirm the changes in *ADK *expression in CRC pathogenesis. According to the published data, tumor tissue shows a local increase in ADK activation, possibly due to adenosine accumulation in the tumor microenvironment and its active metabolism. On the other hand, a decrease in the *ADK-L *mRNA level was observed in the peripheral blood in the group of patients with CRC stage III–IV compared with healthy donors, an inverse relationship was uncovered between the *ADK-L* mRNA levels in cancer patients with tumor extent T2–T4, and the *ADK-S *levels remained unchanged when compared to the controls.

It has been established that some leukocytes populations express CD39/CD73 ectonucleotidases and may be involved in adenosine generation [23], which may lead to immune suppression and tumor growth, particularly in CRC [3, 24]. In this study, we have discovered significant correlations between *CD39 *and the *ADK-S *mRNA levels in the peripheral blood (*r *= -0.468 at *p *= 0.043), as well as *CD73 *and the* ADK-L *mRNA levels (*r *= 0.518 at *p *= 0.0232) in CRC patients. Apart from that, no correlation has been found between the expression level of the *ADK *gene and its isoforms and the changes in the expression levels of the gene coding for the A2aA adenosine receptor, whose activation on lymphocytes boosts immune suppression.

In this paper, for the first time, the relationship between the frequency of the key effector and suppressive lymphocyte populations expressing CD39/CD73 on their surfaces and the changes in the *ADK *expression levels in CRC patients has been analyzed. The analysis of CD4^+^ and CD8^+^ T cells, as well as Treg cell, frequency in the peripheral blood showed that the changes in the CD39+ T cell frequency were most significant in CRC (*Table 3*). For the first time, we have analyzed the relationship between the CD39^+^ and CD73^+^ T cell frequency and mRNA levels for the* ADK *gene and its isoforms in the peripheral blood of CRC patients: No significant correlations were found.

It is currently recognized as a fact that not only T cells can carry CD39 and CD73 ectonucleotidases on their surfaces, but also neutrophils, which are the most common leukocytes in the peripheral blood, B cells, monocytes, and endothelial cells [22, 25]. In this study, RNA for expression analysis was isolated from the whole blood. It is possible that identification of a relationship between the parameters of interest will require a more in-depth assessment with the use of a mononuclear cell fraction (lymphocytes and monocytes) as test material for gene expression analysis and increased sample size.

## CONCLUSIONS

The data obtained in this study and available in the literature show changes in the ADK expression levels in CRC pathogenesis. The relationship between the expression of long and short *ADK *isoforms and the expression of the *CD39*/*CD73 *ectonucleotidases involved in extracellular adenosine generation has been determined. It is indicated that the *ADK-L *mRNA level shows promise as a CRC biomarker. However, no correlation between the expression levels of the* ADK *gene and its isoforms *ADK-L *and *ADK-S *and the contents of CD39/CD73-expressing T cells in the peripheral blood of CRC patients has been found.

## Abbreviations

ADK: adenosine kinase
ADK-L: long isoform of ADK
ADK-S: short isoform of ADK
CD39: ecto-nucleoside triphosphate diphosphohydrolase, ENTPD1
CD73: ecto-5’-nucleotidase, 5’NT
A2aR: adenosine receptor A2a
CRC: colorectal cancer
